# Advances in antibiofilm strategies for medical implant-associated infections: emerging technologies and translational challenges

**DOI:** 10.3389/fmicb.2026.1880016

**Published:** 2026-07-31

**Authors:** Aditi Sharma, Bineypreet Kaur, Aditi Goyal, Purvi, Anu Priya Minhas

**Affiliations:** 1 Department of Biophysics, Panjab University, Chandigarh, India; 2 Department of Biotechnology, Chandigarh College of Technology, Chandigarh Group of Colleges (CGC), Landran, Mohali, Punjab, India; 3 ICMR- National Institute of Malaria Research (ICMR-NIMR), New Delhi, India

**Keywords:** antimicrobial, biofilm, coating, infection, implant, nano

## Abstract

Medical implants are instrumental for modern diagnosis, treatment, and management of disease. However, their increased usage has also raised concerns about implant associated infections. These infections are predominantly caused by microbial biofilms, conferring enhanced resistance to antimicrobial therapies, host immune responses, and environmental stresses, making infections difficult to eradicate. This review provided an updated overview of the evolving antibiofilm landscape for medical implants. It discusses major biofilm forming pathogens and mechanisms of biofilm development, followed by conventional and emerging approaches for biofilm prevention and eradication, including antifouling and antimicrobial coatings, quorum sensing inhibitors, hybrid or multifunctional or combination coatings, antimicrobial peptides, nanotechnology enabled platforms, photodynamic and photothermal therapies, immune targeting strategies, and smart coatings. The review further underscores the challenges associated with these antibiofilm strategies hindering clinical translation. Combating these challenges will play a crucial role in the future, improving antibiofilm therapies and patient outcomes in biofilm related medical implant infections.

## Introduction

Medical implant devices are partly or wholly implanted inside the human body for diagnosis, therapy, and rehabilitation purposes, facilitating disease management, physiological assessment, and restoration of body functions. Catheters, dental, orthopaedic, cardiovascular, and urinary implants are commonly used medical devices (Cao et al., 2022). Dental implants, widely used to restore oral function and aesthetics (Neely and Maalhagh-Fard, 2017), cardiovascular implants, and central venous catheters are essential for medication delivery, hemodynamic monitoring, and cardiac support (Liang et al., 2021) and are vulnerable to bacterial colonization and biofilm establishment, leading to severe complications. The orthopaedic implant market is projected to grow from $46.5 billion to $78.5 billion by 2030 (Chen et al., 2023), highlighting increased reliance on these devices despite persistent infection risks. Majority of implant-associated infections are nosocomial infections and can range from localized to systemic (Haque et al., 2018; He et al., 2021; Kumar et al., 2024; Marina et al., 2022; Jamal et al., 2018). Clinically, polymicrobial biofilms are more abundant, complex, and resilient under dynamic flow than monomicrobial (Joshi et al., 2025), where interspecies quorum sensing (QS) complicates treatment, as targeting a specific pathogen may inadvertently boost another’s virulence (Lamin et al., 2022).

Implant-associated factors, such as liquid flow rate through the device and the presence of host-derived adherent proteins, affect biofilm synthesis (Seneviratne, 2017). Increased implant use has led also raised device-associated infections, as indicated by bloodstream and catheter-related urinary tract infections, compromising patient recovery, increasing costs, and causing implant failure (Mishra et al., 2024; Yadav et al., 2020).

The focus of this review is to highlight the evolving landscape of antibiofilm interventions managing biofilm-associated infections in medical implants. With this focus, the review discusses biofilm-forming pathogens, biofilm development mechanisms, strategies to prevent biofilm formation on medical implants, and clinical translation and regulatory considerations.

### Pathogens associated with biofilm formation

Key pathogens associated with major implant niches include: *Staphylococcus epidermidis* and *Staphylococcus aureus* in cerebrospinal fluid shunts (Fux et al., 2006), *S. aureus*, *Pseudomonas aeruginosa*, and *Pseudomonas acne* on neurosurgical implants like neurostimulators (Conen et al., 2017), and *P. acnes*, *Streptococcus*, *Lactobacillus*, *Bacillus*, and *Mycobacterium* on breast implants (Pajkos et al., 2003). *P. aeruginosa*, *S. aureus*, *S. epidermidis*, *Serratia* spp., *Escherichia coli*, and *Proteus* spp. are reported to cling to contact lenses (Konduri et al., 2021).

Multiple studies have reviewed non-implant-related infection and associated pathogen like dental plaque-associated *Porphyromonas gingivalis*, *Bacteroides forsythus*, and *Actinobacillus actinomycetemcomitans* (Zhao et al., 2023), vaginitis-linked *Gardnerella vaginalis* (Castro et al., 2015); otitis media-associated *Haemophilus influenzae*, *Moraxella catarrhalis,* and *S. pneumoniae* (Otsuka et al., 2013), infective endocarditis by *S. aureus* (Han and Poma, 2022), and necrotizing fasciitis by *Acinetobacter baumannii* (Grier et al., 2021), highlighting that biofilm pathogenesis extends beyond medical device colonization.

### Mechanism of biofilm formation

Physicochemical and environmental stresses, including nutrient starvation, extreme pH, and temperature fluctuations, downregulates motility genes and upregulates adhesion genes triggering planktonic-to-biofilm transition. This transition induces extracellular polymeric substances production, restricting antibiotic penetration, promoting dormant cells, and enhancing long-term survival. Poor sanitation or septicemia can result in biofilm formation on medical implants following systemic inflammatory reaction. Biofilm development is a regulated, multistep process comprising initial adhesion, irreversible attachment, maturation, and dispersion (Figure S1) (Rather et al., 2021). The attachment of bacteria on the implant surface occurs via flagella, pili/fimbriae, adhesins, and non-fimbrial adhesins through physical forces (covalent, hydrophobic, and ionic) and forms permanent bonds with surfaces by secreting EPS consisting of extracellular DNA (eDNA), membrane-derived vesicles, polysaccharides, lipids, and water (Muhammad et al., 2020; Secchi et al., 2022; Campoccia et al 2021; Mugunthan et al., 2023). EPS provides stability, hydration, and protection, whereas eDNA supports biofilm initiation, integrity, and resistance (Sharma et al., 2023; Balducci et al., 2023). EPS accelerates intercellular signalling molecules synthesis like c-di-GMP (Frangleena and Suthindhiran, 2026), stimulating the production of pentasaccharide, alginate, and glucose-rich polysaccharide (Joo and Otto, 2012). Apart from EPS, biofilm maturation is driven by QS, which regulates biofilm formation. QS produces small signalling molecules called autoinducers, which coordinate bacterial group behaviour such as virulence and biofilm formation. Gram-positive bacteria secrete autoinducing peptides, while Gram-negative bacteria secrete N-acyl homoserine lactone as QS molecules (Ruhal and kataria, 2021; Sharma et al., 2023; Jiang et al., 2022). Slowly, as nutrients deplete, mature bacterial colonies disperse from the infection site by upregulating motility (flagella) genes to reattach to a new surface and downregulating surface attachment genes (Kaplan, 2010; Izabel-Shen et al., 2018). However, expression and regulation differ among the different bacterial species. Detachment occurs actively (via nutrient depletion or enzymes) or passively (via fluid shear forces) (Rumbaugh and Sauer, 2020), dispersing cells through abrasion, erosion, and sloughing.

### Strategies to mitigate biofilm formation

Despite antibiotic treatment, biofilm persists during infections. Biofilm-associated microorganisms resist antimicrobial agents and tolerate environmental stress, making conventional treatments less effective. All these factors drive development of thin-film coatings for medical implant surfaces, with research focusing on innovative strategies to prevent biofilm formation or disrupt established biofilms. This review covers conventional anti-biofilm approaches (i.e., antibiofilm and antifouling agents, QS inhibitors (QSI), and hybrid or combination or multifunctional antimicrobial coatings) and Emerging approaches, such as antimicrobial peptides (AMPs), nanotechnology-based, photodynamic and photothermal therapies, bioelectric and acoustic strategies, and smart coatings as summarized in Table 1.

**Table 1:** Summary of advancement in antibiofilm strategies in mitigating implant associated infections.

Category	Strategy	Mechanism of action	References
Conventional techniques	Anti biofilm compounds Antifouling coatings	Inhibit microbial colonization, inhibit biofilm formation, lower microbial adhesion, reduce biofilm biomass by contact- based killing or antimicrobial release.	Campoccia et al. (2013), Su et al. (2025), Durand et al. (2022), Mondal et al. (2021), Saverina et al. (2023), Chen (2022), Li et al. (2024), Wu et al. (2018), Paxton et al. (2021), Rostami et al. (2021), Zhang et al. (2026), Su et al. (2025).
	Inhibition of quorum sensing (QS inhibition coatings)	Regulate biofilm formation by coordinating with global gene expression, disrupt signalling pathways, using natural compounds like polyphenols, flavonoids etc. and synthetic compounds like β-lactams, benzothiazole derivatives etc. which prevent biofilm maturation and persistence.	Juszczuk-Kubiak (2024), Mishra et al. (2024), Naga et al. (2024), Wang Y. et al. (2024), Luo et al. (2017), Wang Y. et al. (2024), Khadraoui et al. (2024), Naga et al. (2024), Naga et al. (2024)
	Hybrid and multifunctional coatings and combination coatings	Generate ROS, inducing oxidative stress with bioelectric and acoustic strategies, damage of cellular components by disrupting membrane integrity. Antimicrobial activity with bioactive elements Increase antibacterial activity, improved penetration into biofilm formation, reduce antibiotic resistance, Interrupt biofilms and boosts treatment efficacy	Sharma et al. (2023), Puspasari et al. (2022), Monika et al. (2025), Yan et al. (2018), Raphel et al. (2016), Mas-Moruno et al. (2018), Wang X. et al. (2025), Wang Y. et al. (2024), Lv et al. (2015), Charria-Girón et al. (2024), Ghosh (2024), Deng et al. (2022), Kaplan et al. (2024), Xinnan et al. (2018), Liu et al. (2026), Fan et al. (2017), Zou et al. (2022), Lee and Kim (2023), Tamimi et al. (2024), Odeh et al. (2025)
Novel Approaches	Novel Antimicrobial peptides coatings	Disrupts microbial membranes, prevents fungal and bacterial biofilm formation provide localized, contact-based activity, limiting early biofilm formation with sustained drug release when immobalized.	Alzahrani et al. (2022), Hashemi et al. (2017), Freitas et al. (2022), Sun et al. (2018), Kazemzadeh-Narbat et al. (2020), Khanda et al. (2025), Riool et al. (2017)
	Nanotechnology based coatings	Reduction in bacterial growth with phage/metal encapsulations for safe penetration, targeting biofilm associated pathogens while still less cytotoxic effects.	Xu et al. (2019), Yi et al. (2020), Singh et al. (2018), Mayorga Ramos et al. (2024), Panthi et al. (2024), Chu et al. (2024), Jayaramudu et al. (2025), Behzad et al. (2020), Kungwani et al. (2024), Pathak et al. (2026), Harun-Ur-Rashid et al. (2025), Panthi et al. (2024), Bouiti et al. (2024)
	Photodynamic therapy Combination (PDT + PTT)	Photo thermal and chemical based antibacterial effect, producing localized heat, ROS production and ion release targeting both planktonic and biofilm associated bacteria.	D’Agostino et al. (2016), Fan et al. (2017), Jia et al. (2019), He et al. (2022), Wang X. et al. (2024), Wang T. et al. (2025), Koç et al. (2025)
	Smart coatings Targeting immune system	Suppress nonspecific protein adsorption. Penetrate mucus barrier, degrade biofilms, generate ROS, Modulate macrophage polarization and immune signalling, Local release of gasotransmitter provides antibacterial efficacy and immune homeostasis exogenous stimulus-responsive therapies	Gallo et al. (2014), Linklater et al. (2020), Kim and Oliver (2023), Su et al. (2022), Xie et al. (2025), Su et al. (2024), Xiu et al. (2023), Wang Z. et al. (2025), Guo et al. (2020), Yang et al. (2024a), Xu et al. (2024), Castanheira and Kubes (2018), Cappelli and Shah (2020), Li et al. (2023), Morandini et al. (2023)
	Stimulus-responsive coating	integrated sensor systems combining antimicrobials at low dose with weak electrical and/or ultrasound waves for real-time detection and monitoring of infections, detects physiological cues- such as pH and temperature shifts, provide “on-demand” release antimicrobial agents only when pathogens are present for infection control.	Joshi et al. (2023), Wang Y. et al. (2024), Xia et al. (2024), Yang et al. (2024a), Albalawi (2024), Wang X. et al. (2024), Pereira (2026), Pal (2024), Hu et al. (2022), Yu et al. (2023)

### Conventional approaches

#### Antifouling, antimicrobial, anti-biofilm coating, and anti-adhesive surface modifications

Since bacterial adhesion and biofilm initiation are influenced by physicochemical surface properties, modifying these properties has become an important strategy to reduce microbial colonization and implant-associated infections (Campoccia et al., 2013; Su et al., 2025). The antimicrobial coatings can eliminate bacteria through contact killing or microbe inactivation, using agents such as metal nanoparticles, polycations and antibiotics (Durand et al., 2022; Saverina et al., 2023). In contrast, antifouling coatings are passive preventive strategies deterring microbes’ settlement on the surface. These include, hydrophilic polymers (polyvinyl alcohol, PVA), poly (2-hydroxyethyl methacrylate) (PHEMA), dextran, and zwitterionic materials reduce protein adsorption and maintain a hydrated interface, minimizing microbial colonization without exerting bactericidal effects (Chen, 2022). Although antifouling strategies prevent initial adhesion, maintaining stable material surface properties is a must, as reviewed in detail by (Li et al., 2024).

The anti-adhesive surface modifications incorporating nanoscale topographies, including nanopillars and nanospikes fabricated from titanium dioxide, graphene oxide, nanoporous polymers, and diamond nanostructures, prevent bacterial adhesion through contact-mediated bactericidal activity. These nanostructures mechanically stretch, deform, or puncture bacterial membranes, causing irreversible membrane disruption and cell death (Wu et al., 2018; Zhang et al., 2024). However, their functionality is limited by challenges such as scalability, cost, and performance under physiological conditions (Su et al., 2025).

#### QS inhibition coatings

QS signalling pathways regulate the expression of genes responsible for virulence, EPS, stress response, and biofilm formation via autoinducers (Mishra et al., 2024; Juszczuk-Kubiak, 2024). Plant metabolites such as polyphenols, flavonoids, phenolics, garlic extract, tea polyphenols, and hamamelitannin are natural non-coating QSIs as reviewed in (Naga et al., 2024). Of these, berberine reports to reduce *S. aureus* adhesion, suppress biofilm development, interfere with QS and lessen the chance of developing antimicrobial resistance (Wang X. et al., 2024). Baicalin, a natural low-toxicity antibiofilm agent, enhances antibiotic efficacy against resistant strains (Wang X. et al., 2024b; Luo et al., 2017). *Myrtus communis phenolic* leaf extract exhibits potent quorum-sensing inhibition, suppressing biofilm formation, swarming, pyocyanin, and protease in *P. aeruginosa* by downregulating QS regulators. In this active extract, among phenolics, 3,5-di-*O*-galloylquinic acid exhibited strongest *in silico* binding to *Chromobacterium violaceum* CviR receptor (Khadraoui et al., 2024).

Antibiofilm strategies eradicate established biofilms using chemical, biological, or physical agents. Synthetic QSIs disrupt autoinducer signaling and biofilm maturation (Naga et al., 2024), whereas surface-functionalized coatings known as coating based QSIs employ DNase I, dispersin B, or lysozyme for degrading the extracellular matrix, promoting biofilm dispersal and enhancing antimicrobial efficacy. Although QSIs reduce bacterial pathogenicity by suppressing QS-regulated virulence factors and exhibit broader applicability, including healthcare, their application is limited by potential resistance development, toxicity, and insufficient clinical validation in humans (Naga et al., 2024).

#### Hybrid, multifunctional, or combination coatings

The above-mentioned strategies, though effective *in vitro* against monomicrobial infections, pose limitations when targeting polymicrobial biofilms (Bayramov and Neff, 2016). Many *S. epidermidis* biofilms resist vancomycin, despite planktonic form is sensitive (Sharma et al., 2023). Hybrid and multifunctional coatings are often interchangeable; hybrid emphasizes the composition of the coating, while multifunctional focuses on functions. ZnO-based hybrid coatings integrated with polymer matrix exhibit antimicrobial and antibiofilm activity via generating reactive oxygen species (ROS) and releasing Zn^2+^ following altered metabolism and membrane integrity, leading to structural damage (Puspasari et al., 2022; Monika et al., 2025; Gocheva et al., 2026). The porous polyetheretherketone hybrid coating embedded with gentamicin and AgNPs (within a silk fibroin matrix) exhibits antibacterial activity via membrane permeabilization, ROS production, DNA damage, and biofilm inhibition compared to sole agent coatings (Yan et al., 2018).

Multifunctional orthopaedic coatings combat infection and promote osseointegration by combining antimicrobial and bioactive agents (Raphel et al., 2016). Micro- and nanoscale engineering favours osteoblast over bacterial adhesion (Mas-Moruno et al., 2018). Bioinspired urushiol polymer is durable, biocompatible, and exhibits antibacterial activity (Wang T. et al., 2025).

Combination therapy augments efficacy by improving penetration and reducing minimum inhibitory concentration through synergistic effects. This includes cumin aldehyde-tobramycin-based enhanced ROS production and biofilm disruption (Wang X. et al., 2024b), and berberine-vancomycin reduces *C. difficile* recurrence (Lv et al., 2015). Further, bacteriophages with vancomycin (Charria-Girón et al., 2024), colistin-based combinations (Wang X. et al., 2024b; Ghosh, 2024), and enzymes like DNase or dispersin B (Deng et al., 2022; Kaplan et al., 2024), show improved biofilm eradication and limit resistance development.

Zwitterionic and poly(ethylene glycol) methacrylate, (PEGMA)-Ti6Al4V coated implant surfaces significantly limit attachment of *S. epidermidis* and *S. mutans* while maintaining biocompatibility with implants (Xinnan et al., 2018). Dopamine-altered polysaccharide sulfate-coated silver-graphene hybrids exhibit a synergistic effect by producing localized heat under near-infrared radiation (NIR) with enhanced Ag^+^ release and antimicrobial effect (Fan et al., 2017). A triple-defense coating of hyaluronic acid, quaternized chitosan, and acylase exhibits anti-adhesion, bactericidal, and quorum-quenching properties (Zou et al., 2022).

Bioelectric effect (BE)-integrated toothbrushes achieve dental implant inflammation by reducing plaque (67%) and bleeding (59%) (Lee, 2023) while achieving ~99% reduction in bacterial biofilms on knee arthroplasty implants (Tamimi et al., 2024). A combination of direct electrical current with chlorhexidine removed >95% vitality of multispecies biofilm from titanium implant compared to chlorhexidine alone (Odeh et al., 2025).

The challenges associated with coatings are clinical translation, toxicity associated with metal nanoparticles and ROS, external stimuli dependence, scalability, production cost, sophisticated material designs, and long-term stability. Therefore, it requires optimization and extensive *in vivo* validation for clinical applications.

### Emerging approaches

#### Antimicrobial peptides (AMPs) coatings

Antimicrobial peptides (AMPs) are surface-active molecules with broad-spectrum activity against implant infections by disrupting microbial membranes, impeding bacterial adhesion, and targeting multidrug resistance. IDR-1018 inhibits biofilms via suppressing (p)ppGpp, while LF-11 reduces biofilm formation and motility (Alzahrani et al., 2022). In preclinical tests, ceragenin CSA-131 prevents fungal and bacterial biofilm formation on endotracheal tubes and exhibits protection against microbial colonization (Hashemi et al., 2017). Self-assembly, polymer conjugation, and nanoparticle integration enhance durability and efficacy (Freitas et al., 2022) (Sun et al., 2018). On surface immobilization, AMPs provide localized, contact-based activity, limiting biofilm formation with sustained drug release (Kazemzadeh-Narbat et al., 2020; Khanda et al., 2025). However, clinical use is limited by scalability challenges, high cost, and regulatory constraints (Riool et al., 2017).

#### Nanotechnology-based coatings

Chloramphenicol-loaded gelatin nanoparticles target biofilms and are effective in reducing bacterial growth by 55.6 and 63.2% in 4 and 8 h, promoting wound healing with minimized cytotoxicity (Xu et al., 2019; Yi et al., 2020). Surface modification can further lower cytotoxicity (Singh et al., 2018). Bacteriophages encapsulated with chitosan or metallic nanoparticles exhibit antibacterial activity and eradicate biofilms while stabilizing and protecting phages, Mayorga Ramos et al. (2024) and Panthi et al. (2024) while TiO₂ nanorods and chitosan-capped TiO₂ hybrids enhance photocatalytic antibacterial activity against *E. coli* and *S. aureus* (Chu et al., 2024; Jayaramudu et al., 2025). Metallic nanoparticles (such as silver, gold, zink oxide (ZnO)) can safely penetrate and target pathogens associated with antibiotic-resistant biofilm matrices with low mammalian cytotoxicity (Behzad et al., 2020; Pathak et al., 2026). Silver-polymer nanocomposites with liposomes show improved antimicrobial efficacy, stability, and suitability (Panthi et al., 2024; Harun-Ur-Rashid et al., 2025). Bouiti et al. (2024) introduced nano-hybrid platforms that integrate nanoscale constituents-such as nanoparticles, nanocomposites, and nanotubes-into polymer matrices to enhance mechanical, optical, and thermal properties while providing advanced self-healing and responsive functionalities. However, it requires standardized safety assessments of such coatings for regulatory approval.

#### Photodynamic and photothermal therapy

Antimicrobial photodynamic therapy (PDT) is a minimally invasive technology employed to eradicate implant-associated multidrug-resistant biofilms. Triangular silver nanoplates designed on glass surfaces exhibit both photothermal and chemical-based antibacterial actions, facilitating the removal of adherent *E. coli* and *S. aureus* biofilms (D’Agostino et al., 2016). The synergistic systems of photothermal agents (PTAs) and antimicrobial agents, like dopamine-altered polysaccharide sulfate-coated silver-graphene hybrids, produce localized heat under NIR, which can enhance Ag^+^ release and microbial death (Fan et al., 2017). PDT represented by cellulose-based coating conjugated with protoporphyrin IX (PpIX) and quaternary ammonium salts significantly upregulate the production of ROS against both forms of bacteria (planktonic and deposited on surfaces) (Jia et al., 2019). PDA/AgNP engineered heterostructures on sulfonated PEEK surface enhance metal ion release with photothermal effects, with efficient antibiofilm activity (He et al., 2022). Wang X. et al. (2024) demonstrate a combination approach involving PDT, photothermal therapy (PTT), and chemodynamic therapy (CDT) to treat cervical tumors (Wang Y. et al., 2024). Wang X. et al. (2025) developed a red-light-activated metal-organic framework (Ce6@ZIF-8-PDA/UBI) employing polydopamine and ubiquicidine peptide, targeting bacteria by penetrating deep into oral biofilm. It helps to achieve 14-day wound healing, eradicates *S. aureus*, *E. coli*, *F. nucleatum*, *P. gingivalis,* and eliminates *S. aureus* biofilms from titanium implants in a mouse model (Wang X. et al., 2025). In another study, both PDT and PTT generated ROS and eradicated biofilms employing heat (Koç et al., 2025). While such systems demonstrate synergistic and targeted destruction of resistant biofilms (utilizing minimally invasive, advanced penetration techniques), their clinical viability is limited by tissue depth, external irradiation, and unmapped host immune responses, with only animal models available rather than human trials.

#### Coatings targeting immune system

Impaired host immunity promotes biofilm survival. Subnanometer implant coating suppresses nonspecific protein adsorption and lowers macrophages/neutrophils, reducing inflammation and promoting tissue regeneration (Gallo et al., 2014; Linklater et al., 2020). Manipulating macrophages restores innate immunity via lncRNA-Cox2 (long non-coding RNA that regulates the cyclooxygenase-2 gene) pathways (Kim and Oliver, 2023); precise regulation is essential to minimize inflammation and impaired tissue integration. Gasotransmitters like hydrogen sulfide and carbon monoxide (CO) exhibit antibacterial and M2-mediated immune homeostasis activities that repair tissue (Su et al., 2022). Metal ion-release coatings modulate immune signals and macrophage polarization: Ag^2+^ and Cu^2+^ favour M2 over M1 macrophages while Zn^2+^ enhances neutrophils and macrophages, suppressing cytokines (Yang et al., 2024a; Zhao et al., 2023). Cryoprotectant CO mitigates inflammation by lowering M1/TNF-/ROS and elevating IL-10 (Wu et al., 2025; Zhou et al., 2024). Probiotic-nanomaterial hybrids (e.g., LP@FeTA/CuPt) combat *H. pylori* by penetrating mucus, generating ROS, preventing biofilms, regulating microbiota, and restoring immunity (Zhang et al., 2026). Depending on exogenous stimuli, stimulus therapies are:

1. Photo Stimulus: With non-invasive PDT and PTT activation, these treatments can destroy targeted cells or pathogens by generating cytotoxic ROS and localized heat, respectively. R-AuSTP-Be platform eradicate biofilms, drive M1-to-M2 polarization, and relieve inflammation (Xie et al., 2025).
2. Ultrasound Stimulus: MnP nanoparticles activated by acoustic energy can trigger cGAS-STING for M1/Th17 responses (Su et al., 2024), while microbubbles improve phagocytosis and M1 polarization (Xiu et al., 2023).
3. Chemodynamic Therapy (CDT): Radical-generating SP-PFe activates M2 polarization (Wang Z. et al., 2025), whereas CuFe_5_O_8_ nanocubes drive M1 polarization and reverse immunosuppression (Guo et al., 2020).
4. Magnetic Hyperthermia: Alternating fields with Mg implants reduce inflammation and promote repair via H^+^ depletion (Yang et al., 2024b) while magnetic nanospheres promote M1 polarization and phagocytise dormant bacteria (Xu et al., 2024).

Immune modulation risks implant tissue damage via sustained release of cytokines, ROS, Neutrophil Extracellular Traps, and enzymes (Cappelli and Shah, 2020; Li et al., 2023).

### Smart coatings

Smart stimulus-responsive coatings represent a transformative shift in orthopaedic implants as self-regulating systems that dynamically manage the implant environment. By detecting physiological cues- such as pH and temperature shifts- these materials provide “on-demand” infection control, releasing antimicrobial agents when pathogens are present. During healthy conditions, they switch functionality to promote osseointegration by facilitating bone cell attachment. By balancing dual roles, these smart coatings optimize the competition between infection prevention and regenerative healing to ensure successful implant integration (Joshi et al., 2023). Smart coatings are integrated sensor systems combining antimicrobials at low doses with weak electrical and/or ultrasound waves for real-time detection and monitoring of infections (Wang et al., 2024). Ultrasound-mediated sonodynamic therapy exhibits antibacterial activity against resistant microbes (Xia et al., 2024) while activated piezoelectric nanomaterials exhibit antibacterial activity against *E. coli* and *S. aureus* by producing electric signals (Yang et al., 2024). Acoustic methods are being explored for non-invasive, real-time biofilm characterization despite challenges (Albalawi, 2024).

Recent stimuli-responsive smart coatings release antimicrobial on-demand, such as pH-responsive titanium coatings, that sustain antibiotic delivery with tissue integration (Pereira, 2026). Pereira (2026) integrated a pH-sensitive poly (methacrylic acid) (PMAA) film on a layer-by-layer (LbL) platform with tetracycline-*β*-cyclodextrin (TCβCD) complex for titanium coating. This demonstrates controlled, pH-dependent drug release, minimizing burst effects at neutral pH while accelerating release in acidic, inflammatory environments enhancing antimicrobial efficacy and promoting favourable soft-tissue integration and collagen maturation without inducing cytotoxicity (Pereira, 2026). Further electrochemical sensing integrated with microfluidics is a cost-effective alternative for low-power bacterial detection. Pal (2024) developed a scalable, cost-effective spray-coating method using a Copper Oxide nanoparticles/TiO₂ hybrid coating on polypropylene surfaces with dual mechanisms of action. The contact killing antimicrobial activity (from CuO nanoparticles) and super hydrophobicity-driven anti-biofouling (from the TiO₂ surface texturing), together achieve a > 6-log reduction (*R* ≥ 6) in bacterial load for both *E. coli* and *S. aureus* while preventing biofilm formation (Pal, 2024).

A titanium-based orthopaedic implant was functionalized with a pH- and NIR-responsive mesoporous polydopamine nanoparticles (MPDA)-luteolin@calcium phosphate coating containing mesoporous polydopamine, the QSI luteolin, and a calcium phosphate shell. Due to acidic biofilm conditions, luteolin is released from the coating to inhibit QS-regulated biofilm formation, while the release of Ca^2+^ and PO₄^3−^ boosts osseointegration. NIR-triggered photothermal activity of MPDA causes bacterial membrane disruption, resulting in protein leakage and ATP depletion. However, this approach requires NIR irradiation, which may damage surrounding tissues, and potential cytotoxicity associated with high concentrations of QSI (Hu et al., 2022). Similarly, a modified titanium implant surface called Ti-polydopamine @S-nitrosoglutathione-osteogenic growth peptide was coated to destroy methicillin-resistant *S. aureus* biofilm, promoting bone healing and osseointegration (Yu et al., 2023).

These coatings are effective, yet their transition to large-scale production is constrained by complex synthesis requirements and microcracking of sol–gel process at higher thicknesses.

### Clinical regulations and translational considerations

Clinical translation barriers to antibiofilm coatings include toxicity, high costs, regulatory hurdles, and limited biofilm penetration efficacy. Additional challenges involve antimicrobial peptide degradation (Mahlapuu et al., 2016) and the requirement of active, long-lasting, and biocompatible nanomaterials or coatings capable of surviving complex *in vivo* environments (Campoccia et al., 2013; Liu et al., 2026). Persistent periprosthetic joint infections demand translatable non-antibiotic antibiofilm strategies (Margaryan et al., 2025). Personalized high-throughput screening of pathogen-specific coatings could improve clinical translation (Ghezzi et al., 2023), similarly to how sodium cocoamphoacetate enhances metronidazole against bacterial vaginosis biofilms better than antibiotics alone (Gottschick et al., 2016) and a combination of electrical current with chlorhexidine in dental implants (Odeh et al., 2025). Translation of nanoparticle-based coatings is limited by cytotoxicity, stability, and delivery issues. Scalability remains difficult because minor particle variations alter efficacy (Kumarage et al., 2026), and excessive immune modulation risks bystander tissue damage or autoimmunity (Van Roy and Kielian, 2025). Consequently, the U. S. Food and Drug Administration and European Medicines Agency approvals require comprehensive pre- and post-market data alongside standardized ISO testing (13,485, 10,993, 14,971) to ensure safety and efficacy (Singh et al., 2026).

## Conclusion

In conclusion, implant-associated biofilm infections remain a major challenge due to the complex and adaptive nature of biofilms, conferring them protection against both host and environmental factors. This review highlights the expanding repertoire of antibiofilm strategies, ranging from conventional to smart responsive coatings. Despite promising preclinical outcomes, the clinical translation of these technologies is constrained by challenges of biocompatibility, cytotoxicity, long-term stability, scalability, and regulatory approval. Furthermore, as implant-associated infections are frequently polymicrobial, future studies must validate emerging strategies using clinically relevant multispecies biofilm models. Future efforts should prioritize clinically relevant polymicrobial models, standardized evaluation methods, and integrated, personalized antibiofilm platforms for improving implant safety, reducing infection rates, and enhancing patient outcomes.

## Supplementary material

SUPPLEMENTARY FIGURE S1 Schematic representation of biofilm development. Biofilm formation proceeds through four sequential stages: initial adhesion, irreversible attachment, maturation, and dispersion (Rather et al., 2021).

## References

[ref1] Albalawi T. (2024). Strategies for detecting bacterial biofilms: unveiling the hidden world of microbial aggregates. Egypt. J. Soil Sci. 64, 1069–1096. doi: 10.21608/ejss.2024.285393.1756

[ref2] Alzahrani N. M. Booq R. Y. Aldossary A. M. Bakr A. A. Almughem F. A. Alfahad A. J. . (2022). Liposome-encapsulated tobramycin and IDR-1018 peptide mediated biofilm disruption and enhanced antimicrobial activity against *Pseudomonas aeruginosa*. Pharmaceutics 14:5. 960. doi: 10.3390/pharmaceutics14050960, 35631547 PMC9144307

[ref3] Balducci E. Papi F. Capialbi D. Del Bino L. (2023). Polysaccharides’ structures and functions in biofilm architecture of antimicrobial-resistant (AMR) pathogens. Int. J. Mol. Sci. 24:4030. doi: 10.3390/ijms24044030, 36835442 PMC9965654

[ref4] Bayramov D. Neff J. (2016). Beyond conventional antibiotics — new directions for combination products to combat biofilm. Adv. Drug Deliv. Rev. 112, 48–60. doi: 10.1016/j.addr.2016.07.010, 27496704 PMC5290225

[ref5] Behzad F. Naghib S. M. kouhbanani M. Tabatabaei S. N. Zare Y. Rhee K. Y. (2020). An overview of the plant-mediated green synthesis of noble metal nanoparticles for antibacterial applications. J. Ind. Eng. Chem. 94, 92–104. doi: 10.1016/j.jiec.2020.12.005

[ref6] Bouiti K. Labjar N. Benmessaoud M. Chraka A. Omari M. Jebbari S. . Nano-hybrid smart coatings: advancements in self-healing and responsive functionalities American Chemical Society, (2024). 279–302.

[ref7] Campoccia D. Montanaro L. Arciola C. (2013). A review of the clinical implications of anti-infective biomaterials and infection-resistant surfaces. Biomaterials 34, 8018–8029. doi: 10.1016/j.biomaterials.2013.07.048, 23932292

[ref8] Campoccia D. Montanaro L. Arciola C. (2021). Extracellular DNA (eDNA). A major ubiquitous element of the bacterial biofilm architecture. Int. J. Mol. Sci. 22:9100. doi: 10.3390/ijms22169100, 34445806 PMC8396552

[ref9] Cao H. Qiao S. Qin H. Jandt K. (2022). Antibacterial designs for implantable medical devices: evolutions and challenges. J. Funct. Biomater. 13:86. doi: 10.3390/jfb13030086, 35893454 PMC9326756

[ref10] Cappelli L. Shah A. (2020). The relationships between cancer and autoimmune rheumatic diseases. Best Pract. Res. Clin. Rheumatol. 34:101472. doi: 10.1016/j.berh.2019.101472, 32029389 PMC7295675

[ref11] Castanheira F. Kubes P. (2018). Neutrophils and NETS in modulating acute and chronic inflammation. Blood 133, 2178–2185. doi: 10.1182/blood-2018-11-844530 30898862

[ref12] Castro J. Alves J. Sousa C. Cereija T. França Â. Jefferson K. K. . (2015). Using an in-vitro biofilm model to assess the virulence potential of bacterial vaginosis or non-bacterial vaginosis Gardnerella vaginalis isolates. Scientific reports, 5, 11640. doi: 10.1038/srep11640 PMC4481526 26113465

[ref13] Charria-Girón E. Zeng H. Gorelik T. E. Pahl A. Truong K. N. Schrey H. . (2024). Arcopilins: a new family of *Staphylococcus aureus* biofilm disruptors from the soil fungus Arcopilus navicularis. J. Med. Chem. 67, 15029–15040. doi: 10.1021/acs.jmedchem.4c00585, 39141525 PMC11403616

[ref14] Chen Z. (2022). Surface hydration and antifouling activity of Zwitterionic polymers. Langmuir 38, 4483–4489. doi: 10.1021/acs.langmuir.2c00512, 35380850

[ref15] Chen X. Zhou J. Qian Y. Zhao L. (2023). Antibacterial coatings on orthopedic implants. Mater. Today Bio 19:100586. doi: 10.1016/j.mtbio.2023.100586, 36896412 PMC9988588

[ref16] Chu G. Guan M. Jin J. Luo Y. Luo Z. Shi T. . (2024). Mechanochemically reprogrammed Interface orchestrates neutrophil bactericidal activity and apoptosis for preventing implant-associated infection. Adv. Mater. 36:e2311855. doi: 10.1002/adma.202311855, 38164817

[ref17] Conen A. Fux C. A. Vajkoczy P. Trampuz A. (2017). Management of infections associated with neurosurgical implanted devices. Expert Rev. Anti-Infect. Ther. 15, 241–255. doi: 10.1080/14787210.2017.1267563, 27910709

[ref18] D’Agostino A. Taglietti A. Grisoli P. Dacarro G. Cucca L. Patrini M. . (2016). Seed mediated growth of silver nanoplates on glass: exploiting bimodal antibacterial effect by nearIR photo-thermal action and ag + release. RSC Adv. 6:70414–70423. doi: 10.1039/C6RA11608F

[ref19] Deng W. Lei Y. Tang X. Li D. Liang J. Luo J. . (2022). DNase inhibits early biofilm formation in *Pseudomonas aeruginosa*- or *Staphylococcus aureus*-induced empyema models. Front. Cell. Infect. Microbiol. 12:917038. doi: 10.3389/fcimb.2022.917038, 36310876 PMC9597695

[ref20] Durand H. Whiteley A. Mailley P. Nonglaton G. (2022). Combining topography and chemistry to produce Antibiofouling surfaces: a review. ACS Appl. Bio Mater. 5, 4718–4740. doi: 10.1021/acsabm.2c00586, 36162127

[ref21] Fan X. Yang F. Nie C. Yang Y. Ji H. He C. . (2017). Mussel-inspired synthesis of NIR-responsive and biocompatible ag-graphene 2D nano-agents for versatile bacterial disinfections. ACS Appl. Mater. Interfaces 10, 296–307. doi: 10.1021/acsami.7b16283 29235842

[ref22] Frangleena S. F. Suthindhiran K. (2026). Pathobiology of ESKAPE biofilms in implant infections: current understanding and implications for future therapeutic strategies. Front. Cell. Infect. Microbiol. 16:1750702. doi: 10.3389/fcimb.2026.1750702, 41878262 PMC13006594

[ref23] Freitas E. Bataglioli R. Oshodi J. Beppu M. (2022). Antimicrobial peptides and their potential application in antiviral coating agents. Colloids Surf. B Biointerfaces 217:112693. doi: 10.1016/j.colsurfb.2022.112693 PMC9262651 35853393

[ref24] Fux C. A. Quigley M. Worel A. M. Post C. Zimmerli S. Ehrlich G. . (2006). Biofilm-related infections of cerebrospinal fluid shunts. Clin. Microbiol. Infect. 12, 331–337. doi: 10.1111/J.1469-0691.2006.01361.X, 16524409

[ref25] Gallo J. Holinka M. Moucha C. (2014). Antibacterial surface treatment for Orthopaedic implants. Int. J. Mol. Sci. 15, 13849–13880. doi: 10.3390/ijms150813849, 25116685 PMC4159828

[ref26] Ghezzi D. Boi M. Sassoni E. Valle F. Giusto E. Boanini E. . (2023). Customized biofilm device for antibiofilm and antibacterial screening of newly developed nanostructured silver and zinc coatings. J. Biol. Eng. 17:18. doi: 10.1186/s13036-023-00326-y, 36879323 PMC9987098

[ref27] Ghosh S. (2024). Polymyxin B plus aerosolized Colistin vs Polymyxin B alone in hospital-acquired pneumonia (‘AEROCOL’ study): a feasibility study. Indian J. Crit. Care Med. 28, 792–795. doi: 10.5005/jp-journals-10071-24767, 39239172 PMC11372667

[ref28] Gocheva Y. Engibarov S. Lazarkevich I. Eneva R. Krumova E. (2026). A review of recent advances in ZnO-enzyme hybrid systems and their applications in the food industry. Science 8. doi: 10.3390/sci8030057, 30654563

[ref29] Gottschick C. Szafranski S. P. Kunze B. Sztajer H. Masur C. Abels C. . (2016). Screening of compounds against *Gardnerella vaginalis* biofilms. PLoS One 11:e0154086. doi: 10.1371/journal.pone.0154086, 27111438 PMC4844189

[ref30] Grier J. T. Arivett B. A. Ramírez M. S. Chosed R. J. Bigner J. A. Ohneck E J. . (2021). Two *Acinetobacter baumannii* isolates obtained from a fatal necrotizing fasciitis infection display distinct genomic and phenotypic characteristics in comparison. Front. Cell. Infect. Microbiol. 11:635673. doi: 10.3389/fcimb.2021.635673 PMC8072282 33912474

[ref31] Guo G. Zhang H. Shen H. Zhu C. He R. Tang J. . (2020). Space-selective chemodynamic therapy of CuFe5O8 nanocubes for implant-related infections. ACS Nano 14, 13391–13405. doi: 10.1021/acsnano.0c05255, 32931252

[ref33] Han J. Poma A. (2022). Molecular targets for antibody-based anti-biofilm therapy in infective endocarditis. Polym. 14:3198. doi: 10.3390/POLYM14153198 PMC9370930 35956712

[ref34] Haque M. Sartelli M. Mckimm J. Abu Bakar M. (2018). Health care-associated infections – an overview. Infect. Drug Resist. 11, 2321–2333. doi: 10.2147/IDR.S177247, 30532565 PMC6245375

[ref35] Harun-Ur-Rashid M. Foyez T. Krishna S. B. N. Poda S. Bin Imran A. (2025). Recent advances of silver nanoparticle-based polymer nanocomposites for biomedical applications. RSC Adv. 15, 8480–8505. doi: 10.1039/d4ra08220f, 40109922 PMC11920860

[ref36] Hashemi M. M. Rovig J. Bateman J. Holden B. S. Modelzelewski T. Gueorguieva I. . (2017). Preclinical testing of a broad-spectrum antimicrobial endotracheal tube coated with an innate immune synthetic mimic. J. Antimicrob. Chemother. 73, 143–150. doi: 10.1093/jac/dkx347, 29029265 PMC5890737

[ref37] He S. Duan C. Wang S. Yu Y. Kei Chan Y. Shi X. . (2022). Fusion peptide-engineered polyetheretherketone implants with photo-assisted anti-pathogen and enhanced angiogenesis for *in vivo* osseointegrative fixation. Chem. Eng. J. 446:137453. doi: 10.1016/j.cej.2022.137453

[ref38] He Y. Li Z. Ding X. Xu B. Wang J. Li Y. . (2021). Nanoporous titanium implant surface promotes osteogenesis by suppressing osteoclastogenesis via integrin β1/FAKpY397/MAPK pathway. Bioact. Mater. 8, 109–123. doi: 10.1016/j.bioactmat.2021.06.033, 34541390 PMC8424426

[ref39] Hu J. Ding Y. Tao B. Yuan Z. Yang Y. Xu K. . (2022). Surface modification of titanium substrate via combining photothermal therapy and quorum-sensing-inhibition strategy for improving osseointegration and treating biofilm-associated bacterial infection. Bioact. Mater. 18, 228–241. doi: 10.1016/j.bioactmat.2022.03.011, 35387171 PMC8961458

[ref41] Izabel-Shen D. Langenheder S. Jürgens K. (2018). Dispersal modifies the diversity and composition of active bacterial communities in response to a salinity disturbance. Front. Microbiol. 9:2188. doi: 10.3389/fmicb.2018.02188 PMC6159742 30294307

[ref42] Jamal M. Ahmad W. Andleeb S. Jalil F. Imran M. Nawaz M. A. . (2018). Bacterial biofilm and associated infections. J. Chin. Med. Assoc. 81, 7–11. doi: 10.1016/j.jcma.2017.07.012, 29042186

[ref43] Jayaramudu T. Varaprasad K. Govil T. Sani R. K. (2025). Chitosan-capped TiO2 hybrid nanomaterials for antibacterial and photocatalytic application of crystal violet. Int. J. Biol. Macromol. 309:142949. doi: 10.1016/j.ijbiomac.2025.142949, 40220833

[ref44] Jia R. Tian W. Bai H. Zhang J. (2019). Sunlight-driven wearable and robust antibacterial coatings with water-soluble cellulose-based photosensitizers. Adv. Healthc. Mater. 8:1801591. doi: 10.1002/adhm.201801591, 30734526

[ref45] Jiang X. Jiang C. Yu T. Jiang X. Ren S. Kang R. . (2022). Benzalkonium chloride adaptation increases expression of the Agr system, biofilm formation, and virulence in *Listeria monocytogenes*. Front. Microbiol. 13:856274. doi: 10.3389/fmicb.2022.856274, 35283841 PMC8905296

[ref46] Joo H.-S. Otto M. (2012). Molecular basis of *in vivo* biofilm formation by bacterial pathogens. Chem. Biol. 19, 1503–1513. doi: 10.1016/j.chembiol.2012.10.022, 23261595 PMC3530155

[ref47] Joshi P. Bhattacharjee R. Sahu M. Gajjar D. (2025). Insights into urinary catheter colonisation and polymicrobial biofilms of Candida- bacteria under flow condition. Sci. Rep. 15:15375. doi: 10.1038/s41598-025-00457-w, 40316568 PMC12048485

[ref48] Joshi M. Kulkarni S. Mounika C. Keerthana M. Kapusetti G. (2023). Current state of art smart coatings for orthopedic implants: a comprehensive review. Smart Mater. Med. 4, 661–679. doi: 10.1038/s41598-025-00457-w

[ref49] Juszczuk-Kubiak E. (2024). Molecular aspects of the functioning of pathogenic Bacteria biofilm based on quorum sensing (QS) signal-response system and innovative non-antibiotic strategies for their elimination. Int. J. Mol. Sci. 25:2655. doi: 10.3390/ijms25052655, 38473900 PMC10931677

[ref51] Kaplan J. (2010). Biofilm dispersal: mechanisms, clinical implications, and potential therapeutic uses. J. Dent. Res. 89, 205–218. doi: 10.1177/0022034509359403, 20139339 PMC3318030

[ref52] Kaplan J. Sukhishvili S. Sailer M. Kridin K. Ramasubbu N., Aggregatibacter actinomycetemcomitans Dispersin B: the Quintessential Antibiofilm Enzyme. Pathogens, (2024) 13:668. doi: 10.3390/pathogens13080668 PMC11357414 39204268

[ref53] Kazemzadeh-Narbat M. Cheng H. Chabok R. Alvarez M. de la Fuente C. Phillips K. S. (2020). Critical reviews in biotechnology strategies for antimicrobial peptide coatings on medical devices: a review and regulatory science perspective strategies for antimicrobial peptide coatings on medical devices: a review and regulatory science perspective. Crit. Rev. Biotechnol. 41, 94–120. doi: 10.1080/07388551.2020.1828810 33070659

[ref54] Khadraoui N. Essid R. Damergi B. Fares N. Gharbi D. Forero A. M. . (2024). *Myrtus communis* leaf compounds as novel inhibitors of quorum sensing-regulated virulence factors and biofilm formation: *in vitro* and in silico investigations. Biofilm 8:100205. doi: 10.1016/j.bioflm.2024.100205, 38988475 PMC11231753

[ref55] Khanda M. Seal P. Mohan A. Arya N. Boda S. (2025). Antimicrobial peptide coatings on implant surfaces – current status and future considerations. Nanoscale 17, 10462–10484. doi: 10.1039/D5NR00953G, 40227869

[ref56] Kim R. Oliver B. (2023). Innate immune reprogramming in COPD - new mechanisms for old questions. Am. J. Respir. Cell Mol. Biol. 68, 470–471. doi: 10.1165/rcmb.2023-0030ED, 36796089 PMC10174163

[ref57] Koç İ. Onbasli K. Kurt C. Atac N. Cooper F. K. Çam K. . (2025). Rational control of combined photothermal and photodynamic therapy for effective eradication of biofilms. Nanoscale 17, 14145–14156. doi: 10.1039/D4NR03798G, 40434218 PMC12118452

[ref58] Konduri R. Saiabhilash C. R. Shivaji S. (2021). Biofilm-forming potential of ocular fluid *Staphylococcus aureus* and *Staphylococcus epidermidis* on ex vivo human corneas from attachment to dispersal phase. Microorg. 9:1124. doi: 10.3390/MICROORGANISMS9061124 PMC8224674 34067392

[ref59] Kumar S. Nasna P. Ghosh G. (2024). Recent advancement in biocompatible materials, hybrid bioactive coating, surface modification and post-processing techniques for the fabrication of biomedical implant: critical review and future prospects. Proc. Inst. Mech. Eng. C J. Mech. Eng. Sci. 239, 3247–3286. doi: 10.1177/09544062241307455

[ref60] Kumarage P. M. Gul T. Green N. J. Wardell S. J. T. Ali M. A. Choudhury M. . (2026). Recent advances in gold and zinc oxide nanoparticles: Antibiofilm action, mechanisms beyond ROS generation, and *in vivo* efficacy. Microbiol. Res. 306:128441. doi: 10.1016/j.micres.2026.128441, 41546960

[ref61] Kungwani N. A. Panda J. Mishra A. K. Chavda N. Shukla S. Vikhe K. . (2024). Combating bacterial biofilms and related drug resistance: role of phyto-derived adjuvant and nanomaterials. Microb. Pathog. 195:106874. doi: 10.1016/j.micpath.2024.106874, 39181190

[ref62] Lamin A. Kaksonen A. H. Cole I. S. Chen X. B. (2022). Quorum sensing inhibitors applications: a new prospect for mitigation of microbiologically influenced corrosion. Bioelectrochemistry 145:108050. doi: 10.1016/J.BIOELECHEM.2022.108050, 35074732

[ref63] Lee J. Kim Y. W. (2023). Bioelectric device for effective biofilm inflammation management of dental implants. Sci. Rep. 13:21372. doi: 10.1038/s41598-023-48205-2, 38049472 PMC10695962

[ref64] Li C. Gao D. Li C. Cheng G. Zhang L. (2024). Fighting against biofilm: the antifouling and antimicrobial material. Biointerphases 19:040802. doi: 10.1116/6.0003695, 39023091

[ref65] Li J. Zhao C. Xu Y. Song L. Chen Y. Xu Y. . (2023). Remodeling of the osteoimmune microenvironment after biomaterials implantation in murine tibia: single-cell transcriptome analysis. Bioact. Mater. 22, 404–422. doi: 10.1016/j.bioactmat.2022.10.009, 36311047 PMC9588995

[ref66] Liang X. Liu Y. Chen B. Li P. Zhao P. Liu Z. . (2021). Central venous disease increases the risk of microbial colonization in hemodialysis catheters. Front. Med. 8:645539. doi: 10.3389/fmed.2021.645539, 34497811 PMC8419307

[ref67] Linklater D. Baulin V. Juodkazis S. Crawford R. Stoodley P. Ivanova E. (2020). Mechano-bactericidal actions of nanostructured surfaces. Nat. Rev. Microbiol. 19, 8–22. doi: 10.1038/s41579-020-0414-z, 32807981

[ref68] Liu X. C. Hu J. Chen X. Yu L. Jia K. Chu Y. . (2026). An antibacterial and antifouling MPC/NaSS/DMA coating against biofilms and thrombi on vascular device implants. Langmuir 42, 8907–8918. doi: 10.1021/acs.langmuir.6c00352 41858202

[ref69] Luo J. Dong B. Wang K. Cai S. Liu T. Cheng X. . (2017). Baicalin inhibits biofilm formation, attenuates the quorum sensing-controlled virulence and enhances *Pseudomonas aeruginosa* clearance in a mouse peritoneal implant infection model. PLoS One 12:e0176883. doi: 10.1371/journal.pone.0176883, 28453568 PMC5409170

[ref70] Lv Z. Peng G. Liu W. Xu H. Su J. (2015). Berberine blocks the relapse of *Clostridium difficile* infection in C57BL/6 mice after standard vancomycin treatment. Antimicrob. Agents Chemother. 59, 3726–3735. doi: 10.1128/AAC.04794-14, 25824219 PMC4468740

[ref71] Mahlapuu M. Håkansson J. Ringstad L. Björn C. (2016). Antimicrobial peptides: an emerging category of therapeutic agents. Front. Cell. Infect. Microbiol. 6:194. doi: 10.3389/fcimb.2016.00194, 28083516 PMC5186781

[ref72] Margaryan D. Dos Santos M. V. Perka C. Trampuz A. Karbysheva S. (2025). Polymicrobial periprosthetic joint infections of the hip and knee. Bone Joint J. 107-B, 1288–1294. doi: 10.1302/0301-620X.107B12.BJJ-2024-1656.R2, 41319694

[ref73] Marina C. Cristina B. Eleonora S. Roberto R. (2022). Environmental, microbiological, and immunological features of bacterial biofilms associated with implanted medical devices. Clin. Microbiol. Rev. 35:e00221-20. doi: 10.1128/cmr.00221-20 PMC8768833 35044203

[ref74] Mas-Moruno C. Su B. Dalby M. (2018). Multifunctional coatings and nanotopographies: toward cell instructive and antibacterial implants. Adv. Healthc. Mater. 8:1801103. doi: 10.1002/adhm.201801103, 30468010

[ref75] Mayorga Ramos A. Carrera Pacheco S. Barba Ostria C. Guaman L. (2024). Bacteriophage-mediated approaches for biofilm control. Front. Cell. Infect. Microbiol. 14:1428637. doi: 10.3389/fcimb.2024.1428637 PMC11491440 39435185

[ref76] Mishra A. Aggarwal A. Khan F. (2024). Medical device-associated infections caused by biofilm-forming microbial pathogens and controlling strategies. Antibiotics 13:623. doi: 10.3390/antibiotics13070623, 39061305 PMC11274200

[ref77] Mondal A. Singha P. Douglass M. Estes L. Garren M. Griffin L. . (2021). A synergistic new approach toward enhanced antibacterial efficacy via antimicrobial peptide immobilization on a nitric oxide-releasing surface. ACS Appl. Mater. Interfaces 13, 43892–43903. doi: 10.1021/acsami.1c08921, 34516076

[ref78] Monika P. Krishna R. H. Hussain Z. Nandhini K. Pandurangi S. J. Malek T. . (2025). Antimicrobial hybrid coatings: a review on applications of nano ZnO based materials for biomedical applications. Biomater. Adv. 172:214246. doi: 10.1016/j.bioadv.2025.214246, 40037050

[ref79] Morandini L. Avery D. Angeles B. Winston P. Martin R. K. Donahue H. J. . (2023). Reduction of neutrophil extracellular traps accelerates inflammatory resolution and increases bone formation on titanium implants. Acta Biomater. 166, 670–684. doi: 10.1016/j.actbio.2023.05.016, 37187302 PMC10330750

[ref80] Mugunthan S. Wong L. L. Winnerdy F. R. Summers S. Bin Ismail M. H. Foo Y. H. . (2023). RNA is a key component of extracellular DNA networks in *Pseudomonas aeruginosa* biofilms. Nat. Commun. 14:7772. doi: 10.1038/s41467-023-43533-3, 38012164 PMC10682433

[ref81] Muhammad M. Muhammad M. H. Idris A. L. Fan X. Guo Y. Yu Y. . (2020). Beyond risk: bacterial biofilms and their regulating approaches. Front. Microbiol. 11:928. doi: 10.3389/fmicb.2020.00928, 32508772 PMC7253578

[ref82] Naga N. Shaaban M. El_Metwally D. M. (2024). An insight on the powerful of bacterial quorum sensing inhibition. Eur. J. Clin. Microbiol. Infect. Dis. 43, 2071–2081. doi: 10.1007/s10096-024-04920-w PMC11534983 39158799

[ref83] Neely A. Maalhagh-Fard A. (2017). Successful Management of Early Peri-Implant Infection and Bone Loss Using a multidisciplinary treatment approach. Clin. Adv. Periodontics 8, 5–10. doi: 10.1902/cap.2017.170014, 32686333

[ref84] Odeh V. Virto L. Garcia-Quismondo E. Herrera D. Palma J. . (2025). Antibacterial effect of combined electrolytic and chemical decontamination methods on dental implant surfaces: *in vitro* study. Clin. Oral Implants Res. 36, 1434–1444. doi: 10.1111/clr.70012, 40772745 PMC12598905

[ref85] Otsuka T. Kitami O. Kondo K. Ota H. Oshima S. Tsuchiya A. . (2013). Incidence survey of acute otitis media in children in Sado Island, Japan-Sado otitis media study (SADOMS). PLoS One 8:e68711. doi: 10.1371/journal.pone.0068711 PMC3699511 23844235

[ref86] Pajkos A. Deva A. K. Vickery K. Cope C. Chang L. Cossart Y. E. (2003). Detection of subclinical infection in significant breast implant capsules. Plast. Reconstr. Surg. 111, 1605–1611. doi: 10.1097/01.PRS.0000054768.14922.44, 12655204

[ref87] Pal S. (2024). Antimicrobial and superhydrophobic CuONPs/TiO2 hybrid coating on polypropylene substrates against biofilm formation. ACS Omega 9, 45376–45385. doi: 10.1021/ACSOMEGA.4C07345, 39554441 PMC11561633

[ref88] Panthi V. K. Fairfull-Smith K. E. Islam N. (2024). Liposomal drug delivery strategies to eradicate bacterial biofilms: challenges, recent advances, and future perspectives. Int. J. Pharm. 655:124046. doi: 10.1016/j.ijpharm.2024.124046, 38554739

[ref89] Pathak A. Singh J. Swati Dwibedi V. (2026). Deciphering microbial biofilm: mechanism, infection, and advanced approaches for control. Folia Microbiologica, 1, 31. doi: 10.1007/s12223-026-01422-4 41758321

[ref90] Paxton W. Paxton W. F. Rozsa J. L. Brooks M. M. Running M. P. Schultz D. J. . (2021). A scalable approach to topographically mediated antimicrobial surfaces based on diamond. J. Nanobiotechnol. 19:458. doi: 10.1186/s12951-021-01218-3, 34963490 PMC8713538

[ref91] Pereira M. M. A. (2026). Targeted infection control and tissue integration via pH-sensitive smart coatings on implant surfaces. ACS Appl. Mater. Interfaces 18:2584–260. doi: 10.1021/acsami.5c19344, 41436403 PMC12781112

[ref92] Puspasari V. Ridhova A. Hermawan A. Amal M. Khan M. M. (2022). ZnO-based antimicrobial coatings for biomedical applications. Bioprocess Biosyst. Eng. 45, 1421–1445. doi: 10.1007/s00449-022-02733-9, 35608710 PMC9127292

[ref93] Raphel J. Holodniy M. Goodman S. Heilshorn S. (2016). Multifunctional coatings to simultaneously promote Osseointegration and prevent infection of Orthopaedic implants. Biomaterials 84, 301–314. doi: 10.1016/j.biomaterials.2016.01.016, 26851394 PMC4883578

[ref94] Rather M. A. Gupta K. Mandal M. (2021). Microbial biofilm: formation, architecture, antibiotic resistance, and control strategies. Brazilian J. Microbiol. 52, 1701–1718. doi: 10.1007/s42770-021-00624-x, 34558029 PMC8578483

[ref95] Riool M. Breij A. Drijfhout J. Nibbering P. Zaat S. (2017). Antimicrobial peptides in biomedical device manufacturing. Front. Chem. 5:63. doi: 10.3389/fchem.2017.00063 PMC5609632 28971093

[ref96] Rostami S. Puza F. Ucak M. Ozgur E. Gul O. Ercan U. K. . (2021). Bifunctional sharkskin mimicked chitosan/graphene oxide membranes: reduced biofilm formation and improved cytocompatibility. Appl. Surf. Sci. 544:148828. doi: 10.1016/j.apsusc.2020.148828

[ref97] Ruhal R. Kataria R. (2021). Biofilm patterns in gram-positive and gram-negative bacteria. Microbiol. Res. 251:126829. doi: 10.1016/j.micres.2021.126829, 34332222

[ref98] Rumbaugh K. P. Sauer K. (2020). Biofilm dispersion. Nat. Rev. Microbiol. 18, 571–586. doi: 10.1038/s41579-020-0385-0, 32533131 PMC8564779

[ref99] Saverina E. Frolov N. Kamanina O. Arlyapov V. Vereshchagin A. Ananikov V. (2023). From antibacterial to Antibiofilm targeting: an emerging paradigm shift in the development of quaternary ammonium compounds (QACs). ACS Infect. Dis. 9, 394–422. doi: 10.1021/acsinfecdis.2c00469, 36790073

[ref100] Secchi E. Savorana G. Vitale A. Eberl L. Stocker R. Rusconi R. (2022). The structural role of bacterial eDNA in the formation of biofilm streamers. Proc. Natl. Acad. Sci. 119:e2113723119. doi: 10.1073/pnas.2113723119, 35290120 PMC8944759

[ref101] Seneviratne C. J. (2017). Microbial Biofilms: Omics Biology, Antimicrobials and Clinical Implications. Boca Raton, FL: CRC Press.

[ref102] Sharma S. Mohler J. Mahajan S. Schwartz S. Bruggemann L. Aalinkeel R. (2023). Microbial biofilm: a review on formation, infection, antibiotic resistance, control measures, and innovative treatment. Microorganisms 11:1614. doi: 10.3390/microorganisms11061614, 37375116 PMC10305407

[ref103] Singh P. Garg A. Pandit S. Mokkapati V. R. S. S. Mijakovic I. (2018). Antimicrobial effects of biogenic nanoparticles. Nanomaterials, 8:1009. doi: 10.3390/nano8121009, 30563095 PMC6315689

[ref104] Singh G. Singh M. Murali A. Gandhi S. Singh O. V. (2026). Dental devices and antimicrobial resistance: challenges, innovations, and regulatory compliances. Frontiers in Medical Technology, 8, 1750006. doi: 10.3389/fmedt.2026.1750006 PMC13047204 41938344

[ref105] Su Z. Kong L. Dai Y. Tang J. Mei J. Qian Z. . (2022). Bioresponsive nano-antibacterials for H2S-sensitized hyperthermia and immunomodulation against refractory implant-related infections. Sci. Adv. 8:eabn1701. doi: 10.1126/sciadv.abn1701, 35394829 PMC8993125

[ref106] Su Z. Xu D. Hu X. Zhu W. Kong L. Qian Z. . (2024). Biodegradable oxygen-evolving metalloantibiotics for spatiotemporal sono-metalloimmunotherapy against orthopaedic biofilm infections. Nat. Commun. 15:8058. doi: 10.1038/s41467-024-52489-x, 39277594 PMC11401848

[ref107] Su Q. Xue Y. Wang C. Zhou Q. Zhao Y. Su J. . (2025). Strategies and applications of antibacterial surface-modified biomaterials. Bioact. Mater. 53, 114–140. doi: 10.1016/j.bioactmat.2025.07.009, 40688018 PMC12274879

[ref108] Sun H. Hong Y. Xi Y. Zou Y. Gao J. Du J. (2018). Synthesis, self-assembly and biomedical applications of antimicrobial peptide-polymer conjugates. Biomacromolecules 19, 1701–1720. doi: 10.1021/acs.biomac.8b00208, 29539262

[ref109] Tamimi I. Gasca M. Halbardier A. Martin S. Martin Caballero G. Lucena Serrano C. . (2024). The treatment of bacterial biofilms cultivated on knee arthroplasty implants using the bioelectric effect. Front. Bioeng. Biotechnol. 12, 1–10. doi: 10.3389/fbioe.2024.1426388, 39015137 PMC11249753

[ref111] Van Roy Z. Kielian T. (2025). Immune-based strategies for the treatment of biofilm infections. Biofilm 9:100264. doi: 10.1016/j.bioflm.2025.100264, 40093652 PMC11909721

[ref112] Wang X. Chen C. Hu J. Liu C. Ning Y. Lu F. (2024). Current strategies for monitoring and controlling bacterial biofilm formation on medical surfaces. Ecotoxicol. Environ. Saf. 282:116709. doi: 10.1016/j.ecoenv.2024.116709, 39024943

[ref113] Wang T. Hou J. Wang Y. Feng X. Liu X. (2025). Urushiol-based antimicrobial coatings: molecular mechanisms, structural innovations, and multifunctional applications. Polymers (Basel). 17:1500. doi: 10.3390/polym17111500, 40508743 PMC12157560

[ref114] Wang Z. Huang Y. He S. Li M. Gong J. Cheng L. . (2025). Oxygen-independent sulfate radical and Fe 2+ −modified implants for fast sterilization and osseointegration of infectious bone defects. ACS Nano 19, 18804–18823. doi: 10.1021/acsnano.5c04147 40350755

[ref115] Wang X. Shi W. Jin Y. Li Z. Deng T. Su T. . (2025). Photodynamic and photothermal bacteria targeting nanosystems for synergistically combating bacteria and biofilms. J. Nanobiotechnology 23:40. doi: 10.1186/s12951-025-03126-2, 39849558 PMC11756032

[ref116] Wang Y. Xu Y. Song J. Liu X. Liu S. Yang N. . (2024). Tumor cell-targeting and tumor microenvironment–responsive nanoplatforms for the multimodal imaging-guided photodynamic/photothermal/chemodynamic treatment of cervical cancer. Int. J. Nanomedicine 19, 5837–5858. doi: 10.2147/IJN.S466042, 38887692 PMC11182360

[ref117] Wu S. Dong R. Xie Y. Chen W. Liu W. Weng Y. (2025). CO-loaded hemoglobin/EGCG nanoparticles functional coatings for inflammation modulation of vascular implants. Regener. Biomater. 12:rbae148. doi: 10.1093/rb/rbae148, 39886364 PMC11781197

[ref118] Wu S. Zuber F. Maniura-Weber K. Brugger J. Ren Q. (2018). Nanostructured surface topographies have an effect on bactericidal activity. J. Nanobiotechnology 16:20. doi: 10.1186/s12951-018-0347-0, 29490703 PMC5830064

[ref119] Xia Q. Jiang H. Liu X. Yin L. Wang X. (2024). Advances in engineered Nano-biosensors for Bacteria diagnosis and multidrug resistance inhibition. Biosensors 14:2. 59. doi: 10.3390/bios14020059, 38391978 PMC10887026

[ref120] Xie J. Huang W. Liu S. Cui Z. Huang B. Tian J. . (2025). Supramolecular Nanoplatform for biofilm eradication and anti-inflammatory by phototherapies and macrophage repolarization. Adv. Healthc. Mater. 14:e2501162. doi: 10.1002/adhm.202501162, 40567056

[ref121] Xinnan C. Murakami T. Tamura Y. Aoki K. Hoshino Y. Miura Y. (2018). Bacterial inhibition and osteoblast adhesion on Ti alloy surfaces modified by poly(PEGMA- r-Phosmer) coating. ACS Appl. Mater. Interfaces 10, 23674–23681. doi: 10.1021/acsami.8b07757, 29944334

[ref122] Xiu W. Li X. Li Q. Ding M. Zhang Y. Wan L. . (2023). Ultrasound-stimulated ‘exocytosis’ by cell-like microbubbles enhances antibacterial species penetration and immune activation against implant infection. Adv. Sci. 11:e2307048. doi: 10.1002/advs.202307048, 38109089 PMC10933665

[ref123] Xu Y. Chen B. Xu L. Zhang G. Cao L. Liu N. . (2024). Urchin-like Fe3O4@Bi2S3 nanospheres enable the destruction of biofilm and efficiently antibacterial activities. ACS Appl. Mater. Interfaces 16, 3215–3231. doi: 10.1021/acsami.3c17888 38205800

[ref124] Xu J. Danehy R. Cai H. Ao Z. Pu M. Nusawardhana A. . (2019). Microneedle patch-mediated treatment of bacterial biofilms. ACS Appl. Mater. Interfaces 11, 14640–14646. doi: 10.1021/acsami.9b02578, 30933463

[ref125] Yadav M. Song J.-J. Singh B. Vidal J. (2020). Microbial Biofilms and human Disease: A Concise Review, Amsterdam, Netherlands: Elsevier B.V. 1–13. doi: 10.1016/B978-0-444-64279-0.00001-3

[ref126] Yan J. Zhou W. Jia Z. Xiong P. Li Y. Wang P. . (2018). Endowing Polyetheretherketone with synergistic bactericidal effects and improved osteogenic ability. Acta Biomater. 79, 216–229. doi: 10.1016/j.actbio.2018.08.037, 30172936

[ref127] Yang N. Wu T. Li M. Hu X. Ma R. Jiang W. . (2024a). Silver-quercetin-loaded honeycomb-like Ti-based interface combats infection-triggered excessive inflammation via specific bactericidal and macrophage reprogramming. Bioact. Mater. 43, 48–66. doi: 10.1016/j.bioactmat.2024.09.012, 39318638 PMC11421951

[ref128] Yang N. Yang X. Cheng S. Gao X. Sun S. Huang X. . (2024b). Magnesium implants with alternating magnetic field-enhanced hydrogen release and proton depletion for anti-infection treatment and tissue repair. Bioact. Mater. 38, 374–383. doi: 10.1016/j.bioactmat.2024.05.010, 38770429 PMC11103218

[ref129] Yang X. Yang Z. Wang X. Guo Y. Xie Y. Yao W. . (2024). Piezoelectric nanomaterials for antibacterial strategies. Appl. Mater. Today 40:102419. doi: 10.1016/j.apmt.2024.102419, 38826717

[ref130] Yi X. Chengyong W. Yu X. Zhishan Y. (2020). A novel bacterial biofilms eradication strategy based on the microneedles with antibacterial properties. Procedia CIRP 89, 159–163. doi: 10.1016/j.procir.2020.05.136

[ref131] Yu Y.-L. Wu J. J. Lin C. C. Qin X. Tay F. R. Miao L. . (2023). Elimination of methicillin-resistant *Staphylococcus aureus* biofilms on titanium implants via photothermally-triggered nitric oxide and immunotherapy for enhanced osseointegration. Mil. Med. Res. 10:21. doi: 10.1186/s40779-023-00454-y, 37143145 PMC10158155

[ref132] Zhang G. Li Z. Sun M. Lu Y. Song J. Duan W. . (2024). Nanostructure-mediated photothermal effect for reinforcing physical killing activity of nanorod arrays. Adv. Sci. 12:2411997. doi: 10.1002/advs.202411997, 39556665 PMC11727397

[ref133] Zhang W. Zhou Y. Li Z. Wang Z. Zhu F. Zou Y. . (2026). Self-actuated probiotic-nanozyme hybrid system with mucus penetration, biofilm eradication and microbiota regulation for *Helicobacter pylori* infection. J. Nanobiotechnology 24:423. doi: 10.1186/s12951-026-04316-2, 41896865 PMC13151254

[ref134] Zhao D. Du C.‐. M. Zuo K.‐. Q. Zhao Y.‐. X. Xu X.‐. Q. Li Y.‐. B. . (2023). Calcium–zinc phosphate chemical conversion coating facilitates the osteointegration of biodegradable zinc alloy implants by orchestrating macrophage phenotype. Adv. Healthc. Mater. 12:2202537. doi: 10.1002/adhm.202202537, 36528867

[ref135] Zhao A. Sun J. Liu Y. (2023). Understanding bacterial biofilms: from definition to treatment strategies. Front. Cell. Infect. Microbiol. 13:1137947. doi: 10.3389/fcimb.2023.1137947, 37091673 PMC10117668

[ref136] Zhou M. Li G. Yu J. Zhou Q. Wang K. Kang J. . (2024). Interfacial delivery of carbon monoxide via smart titanium implant coating for enhanced soft tissue integration with switchable antibacterial and immunomodulatory properties. Bioact. Mater. 40, 318–333. doi: 10.1016/j.bioactmat.2024.06.010, 38978805 PMC11228469

[ref137] Zou Y. Liu C. Zhang H. Wu Y. Lin Y. Cheng J. . (2022). Three lines of defense: a multifunctional coating with anti-adhesion, bacteria-killing and anti-quorum sensing properties for preventing biofilm formation of *Pseudomonas aeruginosa*. Acta Biomater. 151, 254–263. doi: 10.1016/j.actbio.2022.08.008, 35961522

